# Potential Role of Melatonin as an Adjuvant for Atherosclerotic Carotid Arterial Stenosis

**DOI:** 10.3390/molecules26040811

**Published:** 2021-02-04

**Authors:** Rui Zhang, Leng Ni, Xiao Di, Baitao Ma, Shuai Niu, Zhihua Rong, Changwei Liu

**Affiliations:** Department of Vascular Surgery, Peking Union Medical College Hospital, Chinese Academy of Medical Sciences and Peking Union Medical College, Beijing 100730, China; dr_rickyzhang@163.com (R.Z.); nileng@163.com (L.N.); dixiao@pumch.cn (X.D.); 18800158933@163.com (B.M.); niushuai9206@163.com (S.N.); pumc_rzh@student.pumc.edu.cn (Z.R.)

**Keywords:** melatonin, carotid artery stenosis, ischemic stroke, atherosclerosis

## Abstract

Carotid artery stenosis (CAS) is an atherosclerotic disease characterized by a narrowing of the artery lumen and a high risk of ischemic stroke. Risk factors of atherosclerosis, including smoking, hypertension, hyperglycemia, hyperlipidemia, aging, and disrupted circadian rhythm, may potentiate atherosclerosis in the carotid artery and further reduce the arterial lumen. Ischemic stroke due to severe CAS and cerebral ischemic/reperfusion (I/R) injury after the revascularization of CAS also adversely affect clinical outcomes. Melatonin is a pluripotent agent with potent anti-inflammatory, anti-oxidative, and neuroprotective properties. Although there is a shortage of direct clinical evidence demonstrating the benefits of melatonin in CAS patients, previous studies have shown that melatonin may be beneficial for patients with CAS in terms of reducing endothelial damage, stabilizing arterial plaque, mitigating the harm from CAS-related ischemic stroke and cerebral I/R injury, and alleviating the adverse effects of the related risk factors. Additional pre-clinical and clinical are required to confirm this speculation.

## 1. Introduction

Carotid artery stenosis (CAS) is an atherosclerotic disease characterized by the narrowing of the carotid artery lumen due to atherosclerotic plaque formation. Nearly 4.2% and 1.7% of the general population have moderate (narrowing > 50%) and severe (narrowing > 70%) CAS, respectively [1]. As the CAS progresses, the small defects may detach from the unstable and ruptured plaque and could lead to ischemic strokes. New and recurrent strokes severely hinder the prophylaxis of CAS patients. CAS is responsible for around 10% to 20% of ischemic stroke cases [2]. Even for those asymptomatic CAS patients who have no history of stroke or transient ischemic attack, the future stroke risk should not be ignored. Additionally, chronic stenosis of the carotid artery may progress to total occlusion of the artery due to thrombosis. Both unstable plaque and carotid artery thrombosis are the main sources of embolic material in CAS-related ischemic stroke cases.

Atherosclerosis (AS) plays a major role in the etiology of CAS. AS is a pathological process involving inflammation, oxidation, and aberrant immune response, which trigger and fuel excessive infiltration of lipids in artery walls and form arterial plaque. The mechanisms underneath the development of AS are intricate. Chronic inflammation plays one of the significant roles in the process of AS in which the upregulation of a group of pro-inflammatory factors and chemokines, including nuclear factor kappa-B (NF-κB), interleukin-6 (IL-6), IL-1β, tumor necrosis factor-alpha (TNF-α), and the NOD-like receptor family pyrin domain-containing 3 (NLRP3) inflammasome. Oxidative stress is another initiating factor of AS by activating reactive oxygen species (ROS) -generating enzymes, including nicotinamide adenine dinucleotide phosphate (NADPH) oxidases, xanthine oxidases, and cyclooxygenases [3]. The oxidation of low-density lipoprotein (LDL), cytokine secretion, platelet activation and aggregation, and immune cell infiltration into the vessel wall inevitably induce vasodilation of arteries and dysfunction of endothelial and smooth muscle cells [4].

Melatonin (*N*-acetyl-5-methoxytryptamine) is mainly produced as a hormone of the pineal gland in vertebrates [5]. Due to the production of melatonin in mitochondria, where free oxygen radicals occur, melatonin is regarded as a powerful free oxygen radicals’ scavenger that significantly reduces oxidative stress burden [5]. Melatonin is known for its anti-inflammation and immune-regulation properties, and research has shown that melatonin participates in and regulates a variety of pathophysiological processes including apoptosis [6], autophagy [7], mitophagy [8], angiogenesis [9], pyroptosis [10], and aberrant glucose and lipid metabolism [11,12]. Besides its primary use against sleep disorders, melatonin also has vascular protective properties in the contexts of stroke [13], myocardial infarction [14], hypertension [15], heart failure [16], and abdominal aortic aneurysm [17].

Together, due to the potential benefits in cardiovascular diseases and its high safety profile [18,19], melatonin could possibly be an adjuvant for patients with CAS. This review provides an overview of the putative rationale of melatonin used for CAS patients from the angles of prevention of CAS-related risk factors, a potential treatment for CAS, the neuroprotective properties as regards ischemic stroke, and mitigating harm from cerebral ischemia/reperfusion injury that may occur after the revascularization of CAS.

A PubMed search was performed starting from the inception of the database until the present, using the following terms either separately or in combination: ‘melatonin’, ‘carotid arterial stenosis’, ‘vascular’, ‘atherosclerosis’, ‘atherosclerotic plaque’, ‘ischemic stroke’, ‘ischemia/reperfusion injury’, ‘carotid endarterectomy’, ‘smoking’, ‘melatonin’, ‘cigarette’, ‘nicotine’, ‘hypertension’, ‘blood pressure’, ‘hyperlipidemia’, ‘lipoprotein’, ‘oxLDL’, ‘hyperglycemia’, ‘blood glucose’, ‘insulin resistance’, ‘aging’, ‘circadian rhythm’, ‘CLOCK gene’, ‘inflammation’, ‘oxidation’, and ‘stress’. Experimental, clinical, and review articles that covered the relative effects of melatonin were included in this review. Studies of case reports and letters were excluded.

## 2. Pathophysiology of CAS

CAS is a chronic pathological process and remains silent for several decades. The early stage of the CAS could be the deposition of lipids in the subendothelial territory, and the following oxidation and inflammation attract monocyte infiltration and macrophage polarization. Macrophage-derived foam cells can further stimulate the progression of the AS, including endothelial dysfunction, the migration and proliferation of smooth muscle cells, the degradation of extracellular matrix components, calcification, neovascularization, and formation of the fibrous cap and lipid core [20]. Combined with the risk factors of AS, including smoking, hyperlipidemia, and hyperglycemia, the pathological processes of CAS could accelerate, resulting in deterioration of the relatively stable plaque and the remodeling of the carotid artery wall with a narrowed lumen.

It is widely accepted that the vulnerability of the plaque is critical in CAS. A rupture-prone plaque may have several morphological features, including a thin fibrous cap, a large necrotic/lipid core, hemorrhage, calcification, and intraplaque neovascularization, but the specific definition of a plaque vulnerable to CAS varies among different approaches of imaging, i.e., Doppler ultrasound and MRI [21]. The widely accepted idea of vulnerability in CAS usually refers to a high risk of plaque rupture and significant cerebrovascular events [21,22]. The hemorrhagic component exposed to the lumen after plaque rupture and/or the slowing blood flow can induce atherothrombosis. The defects from the plaque and thrombus can flow through the middle cerebral artery and lead to ischemic stroke. Identifying the risk of stroke is critical for the appropriate treatment of CAS patients, but the search for improved therapeutic strategies is ongoing.

## 3. Protective Effects of Melatonin in CAS

In CAS, there are several criteria to identify the severity of disease and the risk of stroke, including the extent of stenosis, intraplaque hemorrhage, a lipid-rich necrotic core, a fibrous cap, luminal area, and the vulnerability of plaque. Although the current guideline regarded stenosis of CAS > 70% as an indication for either surgical or endovascular treatment, recent clinical studies have revealed the potential threat of ischemic stroke in CAS with even moderate stenosis [23]. These findings indicated that the complexity and vulnerability of plaque are also associated with the risk of ischemic stroke. Therefore, the protective effect of melatonin in CAS should be gauged by the anatomic restoration of the carotid artery as well as the stability of the plaque.

Carotid artery intima-media thickness (IMT), which is the distance from the luminal intima to the media-adventitia, has been served as an index for atherosclerotic progression to reflect the risk of cardiovascular events, including carotid artery stenosis. Unlike carotid arterial plaque and stenosis, which refer to more advanced atherosclerosis, IMT increment is not exclusively equal to the early phase of atherosclerosis. IMT increment is an adaptive hypertrophy of the media layer, which could be induced by stimuli including inflammation, infection, or mechanical damage [24]. A previous study has demonstrated that melatonin application significantly reduced carotid IMT by inhibiting cigarette-smoke-induced restenosis [25]. Additionally, there was far less infiltration of CD45-positive inflammatory cells in the carotid artery from rats in the melatonin group, as well as the significantly reduced level of inflammatory cytokines, adhesion molecules, and eNOS [25]. Similarly, melatonin offset the increment in IMT in aorta specimens in an atherosclerosis-prone context induced by a high-cholesterol diet [26]. Although melatonin showed potential anti-atherosclerosis properties in regulating the proliferation, migration, and differentiation of vascular smooth muscle cells and protecting endothelial function in in vitro studies [26,27], more in vivo and clinical evidence is needed to verify the beneficial effect of melatonin in reversing the increment in carotid IMT and reducing the risk of carotid arterial plaque formation.

**Figure 1:**
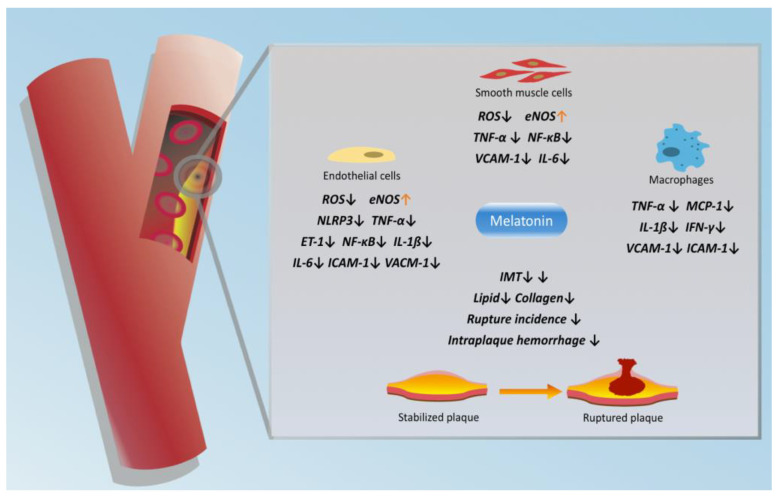
The potential atheroprotective effects of melatonin in attenuating the progression of CAS. eNOS, endothelial nitric oxide synthase. ET-1, endothelin-1. ICAM-1, intercellular adhesion molecule-1. IFN-γ, interferon-γ. IL-1β, interleukin-1β. IL-6, interleukin-6. IMT, intima-media thickness. MCP-1, monocyte chemoattractant protein-1. NLRP3, NOD-like receptor family pyrin domain-containing 3. ROS, reactive oxygen species. VCAM-1, vascular cell adhesion molecule 1.

ApoE^−/−^ mice are used to establish atherosclerotic plaque in the carotid artery by perivascular carotid collar placement [28,29,30]. In a study by Ding et al., the administration of melatonin (10 mg/kg/day, 9 weeks) significantly reduced the intraplaque hemorrhage and plaque rupture incidences in this mouse model with carotid artery plaque [29]. Moreover, mice treated with melatonin had significantly lower flow velocity in the stenotic section of the carotid artery, indicating that melatonin may reduce stenosis in the carotid artery in a plaque-prone context. In another study, only a high dose of melatonin (30 mg/kg/day, 8 weeks) showed a protective effect in mice with carotid artery plaque with reduced vulnerability [30]. However, despite the encouraging results from the above studies, whether melatonin could reverse the plaque formation in the carotid artery is unknown. In terms of molecular mechanism, melatonin administration significantly increased the level of prolyl-4-hydroxylase α1 and collagen in the carotid plaque, whereas the lipid level in the carotid plaque was reduced after the use of melatonin [30]. Additionally, melatonin regulated the macrophage’s polarization and mitigated the inflammation in vulnerable carotid artery plaques via the melatonin-RORα-AMPKα-STAT pathway (Figure 1) [29].

## 4. Melatonin & CAS-Related Ischemic Stroke

Severe CAS influences the intracranial circulation by limiting the inflow of blood to the brain by either severe stenosis or emboli from the unstable atherosclerotic plaques. Ischemic stroke is caused by sudden occlusion of the cerebral artery, which leads to blockage of oxygen and glucose to brain tissue. Previous meta-analysis and clinical studies revealed the potential correlation between CAS and ischemic stroke, in which the presence of carotid plaque and increased carotid intima thickness were associated with the risk of ischemic stroke [31,32]. Ischemic stroke is followed by a vigorous inflammatory response and oxidative stress. Left untreated, inflammatory cascade due to ischemic stroke can lead to a series of biochemical pathologies, including blood–brain barrier damage and brain edema.

Melatonin has been suggested as a potent neuroprotectant for stroke due to the properties of alleviating neuroinflammation and improving brain tissue restoration [13]. Toll-like receptor 4 (TLR4), a membrane receptor for damage-associated molecular patterns, is primarily involved in the inflammation associated with ischemic stroke [33]. TLR4 knockout mice showed a significantly reduced infarcted area than normal mice [33]. In mice with acute ischemic stroke without further reperfusion, melatonin showed an anti-inflammation property by targeting TLR4, which subsequently reduces the expression of inflammatory cytokines of IL-1β/IL-6/TNF-α/IFN-γ/NF-κB as well as the oxidative stress in damaged brain tissue [34]. The study also demonstrated that melatonin administration can significantly reduce infarcted area in the brain both in wild type and TLR4−/− mice. Another study demonstrated that melatonin could alleviate microglial damage by sirtuin 1 (SIRT1) elevation in rats after common carotid artery ligation [35]. In vitro studies showed that melatonin protected and preserved the neuronal cell’s function in the context of hypoxia and low glucose [13,35]. The application of melatonin also preserves the integrity of the blood–brain barrier after transient focal cerebral ischemia due to the inhibition of matrix metalloproteinase 9 (MMP9) [36,37]. Moreover, studies have demonstrated that melatonin application improved cognitive ability and neurobehavior after ischemic stroke in animal experiments [13,38].

The clinical evidence of melatonin in the context of ischemic stroke is limited. In a cohort study, serum level of endogenous melatonin was positively associated with the 30-day mortality of stroke due to large cerebral artery infarction [39]. The elevation of endogenous melatonin in ischemic stroke cases could possibly be explained by the internal protective mechanism against stroke-induced inflammation and oxidative stress, but this hypothetic explanation should be further justified in clinical studies with melatonin application.

## 5. Melatonin & Cerebral Ischemia/Reperfusion Injury

Cerebral ischemia/reperfusion (I/R) injury is a major complication after revascularization of CAS, mainly after surgical revascularization. Patients with cerebral I/R injury typically present prolonged ipsilateral headache without specific intracranial detectable damage [40]. Carotid endarterectomy (CEA) is often used to revascularize severe CAS. As reported by Heo et al., the hyperfusion syndrome rate after CEA was 2.4% (11/452) [41]. I/R injury is associated with extensive production of ROS and induction of oxidative stress, which are direct targets for melatonin. In vivo studies have demonstrated that melatonin used in the animal model of I/R injury can significantly reduce the cerebral infarction volume and improve neurological score, despite the time of drug administration [42,43,44,45]. However, it should be noted that the doses of melatonin used in most of these studies were relatively high (10–50 mg/kg). Mitochondrial dysfunction is a critical pathology in cerebral I/R injury. Wei et al. found that melatonin could modify the defective OPA1-related mitochondrial fusion and protect against cerebral I/R injury [44]. SIRT3, as a potent deacetylase, mainly exists in the mitochondria, and Liu et al. showed that melatonin ameliorates cerebral I/R in a dose-dependent manner via sirtuin 3 (SIRT3) activation [42]. In Zhang et al.’s study, melatonin rendered neuroprotective effects by elevating the expression of RORα, a nuclear receptor known for regulating circadian rhythms and metabolism [45]. Endoplasmic reticulum stress was also to found to be potentially alleviated when melatonin was used in a cerebral I/R injury model [46].

In a randomized controlled trial enrolling 65 patients who underwent CEA due to CAS, 22 patients, who were treated with 6 mg/day melatonin from 3 days before surgery to 3 days after surgery, showed a significantly reduced level of serum inflammatory cytokines and S100β, a protein reversely associated with the extent of brain damage [43]. However, due to the limited sample size and short follow-up, there was no data on the effect of melatonin on the recurrent stroke and other post-operative complications. Although the clinical evidence of melatonin treating CAS-induced cerebral I/R injury is limited, its protective effects of targeted organs against I/R injury in the heart [14], intestine [47], liver [48], and kidney [49] have been demonstrated elsewhere, which indirectly supported the potential benefit of melatonin, alleviating cerebral I/R injury after CAS revascularization.

## 6. Preventive Effects of Melatonin in CAS-Related Risk Factors

The chronic comorbidities, as well as deleterious life habits, are significantly associated with progression of the disease and the deterioration of clinical outcomes of CAS patients. The use of melatonin may mitigate the deleterious effects of the following risk factors in CAS patients.

### 6.1. Smoking

Smoking has become a world pandemic despite years of efforts on cigarette cessation [50]. Smoking is a proven risk factor for various diseases, such as chronic obstructive pulmonary disease, lung cancer, and Alzheimer’s disease. In atherosclerotic diseases, smoking significantly increases the long-term risk of coronary heart disease, stroke, and peripheral artery disease [51]. The multiple system damages caused by smoking may be attributed to the complex mixture of gases produced by tobacco smoking, with more than 4000 chemicals that can be inhaled into the body and adversely affect humans, chronically and systematically. In particular, tobacco smoking contains nicotine, tar, and carbon monoxide, which contribute greatly to atherosclerosis in the arterial system directly and indirectly. In the cardiovascular system, the pathological process of tobacco smoking consists of oxidation, insulin resistance, and sympathetic nerve activation [52]. In addition, smoking is significantly associated with elevated plasma levels of fibrinogen, C-reactive protein (CRP), and IL-6, which refers to an increased inflammatory state in the smoking population. Previous studies suggested that smoking can directly impair the function of the endothelial cell and endothelial progenitor cells of the vascular system [53].

Since smoking fumes contain a group of oxidants and pro-oxidants that can induce free radicals and oxidation, anti-oxidation therapy has long been put forward as a potential treatment for smokers [54]. Investigations over the relationship between endogenous melatonin level and tobacco smoking have been conducted due to the anti-oxidative nature of melatonin. However, the results have not been consistent: compared to non-smokers, Ozguner et al. found a significant decrease in the serum level of melatonin in 21 female smokers [55], whereas Ursing et al. showed a similar level of melatonin in 8 male smokers [56]. However, both studies were limited by a small sample size. Whether smoking induces cardiovascular damage via the suppression of endogenous melatonin production remains unknown.

Further studies over exogenous melatonin supplements against smoking-mediated injury have suggested promising vascular-protective properties. In the animal model, melatonin can reverse the nicotine-induced aorta injury by increasing the expression of endothelial nitric oxide synthase (eNOS) and superoxide dismutase (SOD) and reducing the activity of heat shock protein (Hsp70) and inducible nitric oxide synthase (iNOS) in the vessel wall, which contributes to the reduction of vasoconstriction and oxidative stress [57]. Yang et al. found that melatonin suppressed the expression of a variety of inflammatory cytokines and adhesion molecules in smoke-induced carotid artery stenosis after balloon injury [25]. There was also no significant difference in the intima-to-media ratio of injured arteries between a sham group and a group treated with smoking and melatonin, but the intima-to-media ratio was significantly higher in the smoking group [25]. Another research demonstrated that melatonin significantly increased the plasma level of heme oxygenase-1 (HO-1) and nuclear erythroid 2-related factor 2 (Nrf2) and decreased the level of intercellular adhesion molecule-1 (ICAM-1), vascular cell adhesion molecule-1 (VCAM-1), and endothelin-1 (ET-1) in both animal models and humans [58]. The above findings suggest that melatonin may alleviate smoking-induced endothelial damage via anti-oxidation and anti-inflammation pathways.

### 6.2. Hypertension

Hypertension is second only to smoking as a preventable cause for all-cause mortality [59]. Although more than 90% of hypertension cases defy definite cause, hypertension is a proven risk factor for AS, and hypertension is a strong predictor for future cardiovascular diseases [60]. Hypertension leads to chronic and consistent stress of the vessel wall, contributing to artery stiffness, inflammation, and plaque formation. Hypertension-induced vascular remodeling involves the dysfunction of endothelial cells. In an Ang II-induced hypertension model, the NO and eNOS level of the aortic wall increased significantly [61]. The same study also found the upregulation of the proinflammatory gene of IL-1β, IL-6, and TNF-α in the hypertensive aortic wall. More recent studies suggested that hypertension caused oxidative damage in mitochondria in endothelial cells via SIRT3/superoxide dismutase 2 (SOD2) [62,63]. The findings in the laboratory were confirmed by clinical studies, in which hypertensive patients showed a significant increase in oxidative stress [64,65] and inflammation level [63,66], which could potentially contribute to AS. Although the definite initiating factor of endothelial dysfunction in hypertension is unclear, inflammation and oxidation should play major roles in this process. These findings provide a rationale for the use of melatonin in hypertensive patients.

Endogenous melatonin may positively participate in blood pressure regulation, since rats, after a pinealectomy, were more prone to significant hypertension [67]. Although no clear association between the serum level of melatonin and hypertension was found, patients with hypertension-related cardiovascular diseases were correlated with a reduced level of serum melatonin, which may predict worse clinical outcomes [68,69]. Although melatonin is not a typical anti-hypertensive drug, clinical studies of hypertensive patients found a notable reduction in blood pressure after melatonin treatment [70,71]. Melatonin may attenuate hypertension via the regulation of vasoconstriction and vasodilation and the interplay with the renin-angiotensin system [72]. As reviewed by Pechanova et al., melatonin exerts a hypotensive property in a melatonin receptor-dependent manner or through non-specific pathways [73]. An in vitro study showed that endothelial cells cultured in a high-pressure environment expressed significantly more vasoactive substances, including ET and angiotensin II (Ang II) [74]. Co-incubation of melatonin with endothelial cells demonstrated the downregulation of ET and Ang II and the upregulation of NO production and eNOS expression. Animal studies also showed that melatonin application could attenuate gestational hypertension [75], pulmonary hypertension [76], and neurogenic hypertension [77]. These pre-clinical findings indicated the benefits of melatonin in reducing the tonicity of the vasculature and mitigating hypertension.

Melatonin may also reverse hypertension-induced vascular remodeling. In a study by Simko et al., melatonin application prevented increases in systolic pressure, left ventricular hypertrophy, and aorta wall thickness in a hypertensive rat model by continuous light, an environment with a low secretion of endogenous melatonin [78]. In vascular remodeling induced by pulmonary hypertension, melatonin significantly reduced the expression of remodeling markers (α-actin and smoothelin-B) in resistance vessels [79]. Mechanically, vascular remodeling can also increase the vascular resistance due to the aberrant media-to-lumen ratio and changes in lumen dimensions [80]. However, conflicting results from Quynh N et al. showed that advanced AS may not necessarily result in hypertension in ApoE^-/-^ mice [81]. Despite the regulatory mechanism of melatonin in hypertension, sufficient evidence has indicated the potential hypotensive properties of melatonin.

### 6.3. Hyperlipidemia

Hyperlipidemia is associated with increased risk of AS, and the treatments that lowering serum lipids can reduce cardiovascular risk [82]. Hyperlipidemia refers to an aberrant increase in LDL, total cholesterol, triglyceride levels, or lipoprotein levels or a decreased level of high-density lipoprotein. Low-density lipoprotein is a well-recognized pathogenic factor that is susceptible to oxidation and prone to retention in the subendothelial space, contributing to the formation of inflamed atherosclerotic plaques. Oxidized LDLs (oxLDLs) can induce a series of pathophysiological processes in endothelial cells, including proliferation [83], apoptosis [84], pyroptosis [85], and inflammation [86]. Dysfunction of endothelial cells caused by oxLDLs is critical. oxLDL uncouples the efflux of cholesterol from endothelial cells and augments the production of ROS by NLRP3 inflammasome pathway activation, which is a potential pathway leading to AS [87,88]. The atheroprotective role played by HDL was indicated by an inverse correlation between HDL level and cardiovascular events [89,90]. HDL regulates tissue cholesterol homeostasis by transporting excessive lipids from peripheral tissue to the liver for further metabolism [91]; moreover, HDL, as an antioxidant, effectively prevents lipid oxidation [92]. The HDL’s capacities for anti-inflammation and immune activation also enhance endothelial function [93].

A potential reversed correlation between serum levels of endogenous melatonin and oxLDL was found in patients with AS-related disease [94]. Experimental studies further confirmed that melatonin, through oral intake or intraperitoneal injection, significantly reduce the plasma levels of LDL and VLDL and increase the level of HDL in high cholesterol diet rats and rats with metabolic disorder [95,96,97]. A meta-analysis of eight randomized controlled trials showed that melatonin application significantly reduced the level of triglycerides, total cholesterol, and LDL in peripheral blood [98]. However, the results were limited by a small sample size, a potential bias from the heterogeneity in patients’ underlying diseases, and different melatonin doses among studies. Hepatocytes play an essential role in serum lipid regulation and consumption. In hepatocytes, melatonin showed a protective effect via the SIRT1/mitofusin2 pathway to reduce ROS production in the context of lipid-induced mitochondrial dysfunction and hepatic fibrosis [99]. In vitro studies also indicated the potential benefit of melatonin use in the metabolic function of hepatocytes. Pretreatment of melatonin in HepG2 cells showed an improved consumption of lipids and an upregulation of PPARα and carnitine palmitoyl-CoA transferase 1 (CPT1), both of which are lipolytic genes essential for lipid metabolism [100]. The LDL receptor is a crucial regulator of plasma lipids, and LDL-receptor-deficient animals have long been used as AS models [101]. Ma et al. used melatonin to prevent the downregulation of LDL receptors induced by cigarette smoke exposure in HepG2 cells [11]. An in vitro study conducted by Song et al. showed that melatonin was protective of hepatocytes against inflammation-induced damages by reducing mitochondrial stress and Akt-SIRT3 pathway activation [102].

Melatonin’s property against the oxLDL-induced endothelial damage has been well documented. Zhang et al. found that melatonin reversed the oxLDL-induced pyroptosis in endothelial cells via the MEG3/miR-223/NLRP3 axis [103]. Notably, oxLDL leads to inflammasome activation and pro-inflammatory factor secretion, and melatonin treatment significantly reduced the production of NLRP3 inflammasome and IL-1β secretion in oxLDL-treated macrophages [104]. Although results of a meta-analysis did not show a significant increase in HDL with the use of melatonin [98], an in vivo study of rats suggested that melatonin may potentially elevate HDL serum levels, especially in rats with hyperlipidemia induced by high fructose diets [96] and pre-diabetic rats [105]. In another supportive study by Santos et al., compared to normal rats, pinealectomized rats have significantly reduced serum levels of HDL, while pinealectomized rats with melatonin supplement demonstrated increased serum levels of HDL [106]. Still, the correlation between HDL and melatonin is unclear due to inconsistent results, and the effect of melatonin on HDL functionality is warranted. In general, melatonin’s properties of hepatocytes protection and the lowering of circulating lipids showed the potential therapeutic value of melatonin in improving lipid regulation and thus mitigated the process of AS.

### 6.4. Hyperglycemia

Hyperglycemia is a result of carbohydrate metabolism deficit associated with a group of pathologies, including diabetes mellitus, insulin resistance, and glycogen synthesis disorder. Moreover, the crosslinks among hyperglycemia [107], inflammation, oxidation stress [108], and hyperlipidemia play a critical role in the AS process. Numerous clinical studies showed that patients with hyperglycemia are more prone to AS-related diseases [109], and blood glucose therapies effectively prevent cardiovascular diseases [110]. Hyperglycemia-linked endothelial dysfunction may correlate with increased vascular permeability, upregulation of adhesion molecules, chemokines, and ROS [111,112]. Hyperglycemia also contributed to the atherogenic modification of LDL and triggered the pathological accumulation of lipids in AS plaque [113]. The chronic inflammation and oxidation induced by hyperglycemia are triggered by pathways including the AGEs-RAGE axis, the polyol pathway, and PKC activation [112].

Melatonin participates in the regulation of blood glucose. In animal studies, a pinealectomized model showed the exacerbation of blood glucose control in the context of autoimmune diabetes [114,115] and type 2 diabetes [116], while extra treatment of melatonin attenuated the progression of the disease and protected the animal. In a human study, McMullan et al. demonstrated a reversed correlation between melatonin level and the incidence of type 2 diabetes in humans, in which lower melatonin secretion was independently associated with a higher risk of type 2 diabetes [116]. The nucleotide polymorphisms that uncoupled the melatonin receptor were also correlated with type 2 diabetes hazards, further indicating the beneficial role of melatonin in blood glucose regulation [117]. In a study by Sartori et al., melatonin supplement augmented the insulin sensitivity and glucose tolerance in mice with a high-fat diet [118]. Interestingly, melatonin did not show the same benefits in mice with a normal diet [118]. Similarly, Li et al. did not find a beneficial effect of melatonin in a controlled group of rats in terms of blood glucose level, HbA1c, serum insulin level, and β cells in the islets [119]. However, in a smoking group, melatonin significantly ameliorated smoking-induced hyperglycemia and improved hepatic glycogen synthesis [119]. In a Zucker diabetic fatty rat model, which has a higher initial weight and weight gain, melatonin showed lipid homeostasis regulation in the context of obesity and hyperglycemia [120]. Additionally, melatonin exerts benefits in other hyperglycemia-induced pathological models, including cardiac apoptosis [121], myocardial I/R injury [122], cardiac fibrosis [123], and retinopathy [124]. These findings further confirm the atheroprotective benefits of melatonin in the context of hyperglycemia.

### 6.5. Aging

Aging, a common risk factor for cardiovascular diseases, is related to the physiological process of decline in the organelle, cells, organs, and related functions. Aging-related cardiovascular disease is a massive burden for the aged population. Atherosclerosis is regarded as a biomarker of biological vascular aging, which participated in a group of aging-related cardiovascular diseases such as stroke, myocardial infarction, carotid artery stenosis, and hypertension [125]. During biological vascular-aging, physiological change in morphology in terms of arterial stiffening occurred due to the replacement of elastin fibers with collagen fibers [126]. This may explain the higher risk of hypertension in the aged population, which can precede the progression of AS. Additionally, aging is coupled by reduced aortic vasorelaxation capacity and increased vascular susceptibility to pathogens [127].

Aging is correlated with endothelial dysfunction, in which pro-inflammatory state and pro-oxidative state occurred and induced senescence, apoptosis, and necrosis of endothelial cells [128]. Mitochondria damage is associated with atherosclerosis by impairing mitochondrial DNA, endothelial dysfunction, smooth muscle cell differentiation, and macrophage activation [129]. Previous studies supported the theory that ROS accumulation in mitochondria played a critical role in the aging process on the molecular level, and ROS can activate PARP1, upregulate pro-inflammatory cytokines, and impair mitochondrial integrity [130]. Aging is also coupled by telomere attrition, which is associated with the development of cardiovascular diseases and the progression of atherosclerosis [131]. Moreover, pathological factors, including oxidative stress and chronic inflammation, can precede the progression of telomere shortening, the vicious circle of which contributes to the aging of the vasculature system [132]. Clinical studies indicated that AS-related vascular diseases progressed along with the aging process, which increases the risk of cerebrovascular diseases, coronary artery disease, and peripheral arterial diseases [133].

Melatonin is regarded as an anti-aging agent in a variety of pathological models, such as age-related impairments in the prostate [134], bone [135], ovaries [136], and brain [137]. The anti-aging property of melatonin is correlated with the protective role of melatonin against mitochondrial dysfunction. In particular, age-related mitochondrial dysfunction induces ROS and reactive nitrogen species (RNS), while melatonin can effectively reduce ROS/RNS generation [138]. Melatonin also plays the role of a scavenger of free radicals to inhibit the oxidative stress in mitochondria. Recent studies demonstrated that melatonin can alleviate mitochondrial oxidative stress-induced via SIRT3/SOD2 pathway, though the experimental models were not aging-related [139,140]. Another therapeutic target for aging by melatonin application is inflammation. Volt et al. demonstrated that aging activated NF-κB and NLRP3 inflammasome, both of which were potential targets of melatonin [141]. In a study by Volt et al., melatonin supplement in aged mice showed a reduction in inflammation and oxidative stress level and mitochondrial function enhancement [141]. The effect of melatonin on the aging-associated inflammatory network was critically reviewed by Hardeland et al. in a recent publication [142].

Studies have suggested the beneficial effect of melatonin in aging-related vascular damage. In a study by Rodella et al., 6- and 15-week-old apolipoprotein E-deficient mice were treated by oral intake of melatonin, and the results suggested that melatonin improved age-related endothelial dysfunction via the SIRT1-p53-eNOS axis [143]. Lee et al. demonstrated melatonin endothelial function and integrity against the aging process by applying melatonin in young (3-month-old) and aged (8-month-old) groups of mice for comparison [127]. In this study, the vascular damage was significantly severe in all three pathological models: the critical limb ischemia model, the carotid artery wire injury model, and the aortic injury model. Melatonin application vastly alleviated the intimal/medial hyperplasia in the carotid artery, aortic injury, and improved blood flow in the critical limb ischemia model. The results suggested that the SIRT signaling pathway may play a critical role in the protective effects of melatonin in aging-related vascular impairment. However, there is a shortage of pre-clinical and clinical studies of specific aging-related AS models treated by melatonin application. Considering the potential benefit of melatonin on other diseases induced by aging and aging-associated endothelial impairment, melatonin might be an adjunct agent against aging-related AS.

### 6.6. Disrupted Circadian Rhythm

Circadian rhythm refers to an intrinsic timekeeping mechanism that controls the rhythm due to day/night cues in the human body. There is credible evidence to suggest that circadian rhythm is highly related to the cardiovascular system, including heart rate, blood pressure, and platelet function. Additionally, circadian clocks are found in cardiomyocytes, vascular smooth muscle cells, and endothelial cells. Experimental findings indicated that misalignment of the day/night cycle induces aberrant pathophysiology in cardiomyocytes and vascular smooth muscle cells [144]. There is clinical correlation between disrupted circadian rhythm and adverse cardiovascular events, as well as related risk factors, including diabetes mellitus, hyperlipidemia, and hypertension [145,146]. A circadian rhythm disorder can induce the mutation of core clock genes in cells as well as an inflammatory condition by the increased level of pro-inflammatory cytokines due to immune system stimulation [145]. Additionally, circadian rhythm contributes to the change and modulation in the complex euroendocrine system, which is synchronized with the circadian clocks in both the suprachiasmatic nucleus and peripheral tissues [19].

Experimental findings suggested that circadian-related genes are involved in the progression of CAS. The circadian gene CLOCK has protective effects against atherosclerosis. CLOCK over-expression attenuates the endothelial damage induced by high glucose [147]. Moreover, knockout of the CLOCK gene in mice with carotid artery ligation increases neointima formation and neovascularization [148]. In a human carotid plaque sample, the expression level of CLOCK was also negatively correlated with plaque vulnerability, as well as the presence of atherosclerotic risk factors [147,148]. Another circadian gene, brain and muscle Arnt-like protein-1 (BMAL1), plays an essential role in circadian rhythms, the loss of which can cause abnormality including aging, aberrant blood glucose level, shortened longevity, and aggravated atherosclerosis [149]. Similarly, BMAL1 expresses significantly in the carotid artery with ligation injury [150]. In a clinical study, Obayashi et al. showed that prolonged and more intense exposure of light at night, which adversely affected circadian rhythms, was correlated with a significant increase in IMT of the carotid artery [151]. Notably, the results of the study suggested that disrupted circadian rhythms induced by extensive light exposure are an independent risk factor for subclinical carotid atherosclerosis [151].

Melatonin, as an euroendocrine hormone, is secreted in accordance with the variation of the circadian clock. Previous studies have shown that melatonin receptor MT1 was required for regulation of the circadian gene in peripheral tissue by melatonin application [152,153]. However, there is inconsistency regarding the role of the MT2 receptor in circadian regulation [153,154]. A clinical study showed that circadian misalignment induced by increased light exposure could suppress plasma melatonin concentration [155]. The application of melatonin also showed protective effects in cardiovascular pathology through the circadian rhythm pathway [156]. In a mouse model of atherosclerotic CAS, the intake of melatonin significantly lowered the incidence of intraplaque hemorrhage and plaque rupture by involving the regulation of RORα, a circadian nuclear receptor [29]. Melatonin also regulated the circadian gene CLOCK and BMAL1 in the pathology of hepatocellular carcinoma [157], sepsis [158], and diabetes mellitus [159]. However, whether melatonin alleviates atherosclerosis through CLOCK and BMAL1 regulation is unknown. Taken together, melatonin application may benefit CAS patients by alleviating the adverse effects of a disrupted circadian rhythm.

## 7. Clinical Implications and Limitations

**Figure 2:**
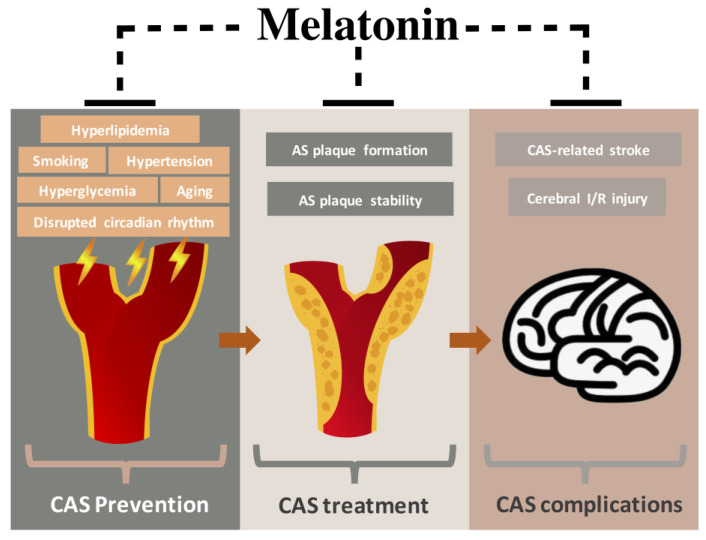
Illustration of the potential preventive and therapeutic properties of melatonin in atherosclerotic carotid artery stenosis. AS, atherosclerosis; CAS, carotid artery stenosis; I/R, ischemia/reperfusion.

This review provides an overview of the potential preventive and therapeutic effects of melatonin against CAS (Figure 2). In this review, the protective effect of melatonin in the five essential risk factors for CAS, including smoking, hypertension, hyperlipidemia, hyperglycemia, and aging, is thoroughly discussed. Since CAS is a chronic pathological process, the alleviation of risk factors could be useful in preventing CAS in the first place and in mitigating CAS progression. In the assessment of carotid IMT, which is normally regarded as the early stage of atherosclerotic plaque, melatonin may reverse the increment of carotid IMT, but the related studies are limited, and there is a lack of verification from clinical studies. As for carotid arterial plaque, melatonin may enhance its stability, which is crucial in reducing the rate of stroke. A variety of studies have demonstrated the neuroprotective properties of melatonin in both stroke animal models and patients with stroke. Therefore, melatonin administration may reduce the cerebral damage and restore neural function when ischemic stroke attacks CAS patients.

However, the above rationales for melatonin as an adjuvant drug in CAS patients are based on scattered research and logical inferences in related fields. Whether melatonin can elicit a beneficial effect on patients with CAS requires more sophisticated pre-clinical and clinical studies. Notably, melatonin may demonstrate variable effects under different conditions and in different cell types [160]. There is evidence to suggest that melatonin also renders pro-inflammatory effects in basal or suppressed immune conditions [161]. Thus, whether melatonin is effective and safe in CAS patients with different immune conditions and inflammation states is unknown. Moreover, the molecular mechanisms behind melatonin’s atheroprotective effects are yet to be unveiled. Although the current knowledge about melatonin indicates the potential benefits of medical, endovascular, and surgical treatments in patients with a high risk of CAS and a related threat of stroke, there is a lack of evidence that the long-termed administration of melatonin can reduce the risk of CAS or even reverse carotid plaque progression. Whether melatonin can interact with other atheroprotective drugs (e.g., lipid-lowering drugs and anti-platelet drugs) is also under-reported.

## 8. Conclusions

Pre-clinical and clinical evidence suggests the potential benefits of melatonin in preventing and treating atherosclerotic CAS, CAS-related risk factors, and CAS-related complications. However, due to a lack of studies with a sophisticated design and an appropriate sample size, there is no high-quality evidence supporting the use of melatonin with CAS patients. Further experimental verification is needed to better understand the application of melatonin in CAS patients and future clinical transformation.

## Data Availability

Data sharing is not applicable.
